# Health effects of heating, ventilation and air conditioning on hospital patients: a scoping review

**DOI:** 10.1186/s12889-020-09358-1

**Published:** 2020-08-26

**Authors:** Benedikt Lenzer, Manuel Rupprecht, Christina Hoffmann, Peter Hoffmann, Uta Liebers

**Affiliations:** 1 Department of Outpatient Pneumology, Charité – Universitätsmedizin Berlin, corporate member of Freie Universität Berlin, Humboldt-Universität zu Berlin, and Berlin Institute of Health, Berlin, Germany; 2 Institute of Laboratory Medicine, Clinical Chemistry, and Pathobiochemistry, Charité – Universitätsmedizin Berlin, corporate member of Freie Universität Berlin, Humboldt-Universität zu Berlin, and Berlin Institute of Health, Berlin, Germany

**Keywords:** HVAC, Electrical fan, Health facility environment, Hospital design, Climate change, Temperature, Heat, Cold, Patient comfort, Patient outcomes

## Abstract

**Background:**

In the face of climate change, the protection of vulnerable patients from extreme climatic conditions is of growing interest to the healthcare sector and governments. Inpatients are especially susceptible to heat due to acute illness and/or chronic diseases. Their condition can be aggravated by adverse environmental factors. Installing air conditioning can be seen as an element of public health adaptation because it was shown to improve mortality rates of hospital patients experiencing hot temperatures. Still, the mediating factors and resulting health effects are largely unknown.

**Method:**

The PRISMA-ScR guideline was followed for this scoping review. Available evidence on the health effects of Heating, Ventilation, Air Conditioning (HVAC) and fans was searched in Medline, Embase and the Cochrane Library. The focus of the search strategy was on inpatients of the hospital. Grey literature was screened on 14 relevant websites. English and German publications were eligible without restrictions on publication date. Results were charted according to the categories population, intervention, control and outcome together with a qualitative description.

**Results:**

The review process yielded eleven publications of which seven were issued after 2003. Seven were clinical trials, three cross-sectional studies and one was a case report. The publications described the installation of HVAC on general wards and in intensive care units. Main topics were heat stress protection and support of thermoregulation, but also the rewarming of hypothermic patients. HVAC use resulted in a recovery effect shown by improved vital signs, reduced cardiac stress, accelerated recuperation and greater physical activity. This protective effect was demonstrated by a shorter hospital stay for patients with respiratory disease and a reduction of mortality for heat illness patients.

**Conclusion:**

This scoping review summarises the fragmented evidence on health effects of HVAC and fan utilisation for inpatients. Installing HVAC has the potential to improve patients’ outcomes and to make hospital treatment more efficient during heat waves. The application of HVAC could be a promising adaptation measure to mitigate the adverse effects of climate change on health and healthcare systems.

## Background

As global warming has become a reality, the mitigation of climate change and the creation of a climate change resilient healthcare system have become essential topics [1–4]. The World Meteorological Organization confirmed the continuing “long-term warming trend” based on five independently collected global datasets [5, 6]. The increasing number of severe weather events, like heat waves, cold spells, extreme rainfalls or storms, will particularly negatively affect cities and hence stress the healthcare infrastructure typically found in urban areas [1, 2, 7]. One result is a rise in mortality and morbidity in heat-sensitive populations. Vulnerable groups include paediatric and adult patients with cardiovascular, renal or pulmonary disease but also patients with geriatric syndromes, heat-sensitive medication or bedridden individuals [1, 2, 8–13]. A meta-analysis showed that patients with chronic lung disease will have an increased mortality of at least 1.8% due to future heat waves [9]. A similar effect was also seen with hospital patients [14]. Additionally, reports on negative outcomes are emerging in disciplines like surgery or psychiatry which are often not in the centre of adaptation efforts [15, 16].

To this day, adaptation endeavours such as installing heat-health action plans remain behind the goals of the Paris Agreement of 2016 [2]. As infrastructure is the cornerstone of healthcare systems, explicit focus of hospitals on climate change-preparedness is necessary [2]. Installation of Heating, Ventilation, Air Conditioning (HVAC) in medical facilities is uncommon in Central and Northern Europe compared to Southern Europe or North America. This impairs the ability of these healthcare systems to cope with more frequent peaks of extreme weather conditions.

### What is known of the health effects of heating, ventilation and air conditioning?

The current knowledge of HVAC related effects is based on studies on health issues in office workers [17] or on exercise-related heat illness [18, 19]. Climatic influences on the physiology were tested with volunteers in climatic chambers [20, 21]. Moreover, many publications are concerned with the spread of infections [17, 22].

Equipping hospital rooms with some form of mechanical ventilation or air conditioning is one method to safeguard patients during heat or cold periods. The goal hereby is to ensure the quality of medical care and thermal comfort for patients and staff alike. An array of HVAC-systems is available, ranging from air conditioners or chillers to radiant cooling systems and fans. The protective effect of HVAC was demonstrated by a mortality reduction in private homes and hospitals [23–25]. However, since the examined studies were incoherent, a Cochrane review of the use of electrical fans did not yield conclusive results for or against the use in the general population [26].

Overall, studies on the effects of air conditioning on the health of inpatients are scarce. Knowledge of the mechanisms leading to effects on patient health when using HVAC is particularly insufficient. Hence, the first objective was to systematically screen the evidence on HVAC use and associated clinical effects or health-related outcomes in inpatients. The second objective was to identify relevant findings to generate new hypotheses for research on the efficacy of HVAC in climate change adaptation.

## Methods

This research project was carried out according to the PRISMA Extension for Scoping Reviews (PRISMA-ScR) [27]. After a first non-systematic search, study registries were screened. One review project related to the topic could be identified (Prospero-ID: CRD42015018970), but progress status and email contact revealed no current activities. The protocol of our scoping review was registered on www.researchregistry.com (UIN: reviewregistry675).

### Eligibility criteria

Literature on adult inpatients in English or German language was considered. The aim was to identify publications which reported health effects of all types of HVAC. Evidence on heating and cooling was included since many systems can technically be applied for both. Above all, through climate change different kinds of weather extremes will become more frequent. Articles that described direct body cooling methods such as fanning the body from a near distance were excluded. Literature on HVAC in the operating room or on infection rates was not within the scope of this review. Passive cooling interventions like window sunscreens were not included. Any date of publication was eligible to obtain a thorough overview of the published literature.

### Search process

#### Database search

Medline, Embase and the Cochrane Library were searched. Within the search strategy of this review, the emphasis was put on clinically relevant parameters for internal medicine and pulmonology. Hence, search terms containing morbidity and mortality were not included in the search strategy. The full search strategy can be found in Additional file 1. The database search was carried out in April 2019.

#### Grey Literature & Other Sources

14 websites of technical, medical and governmental organisations were screened (for web addresses see Additional file 2). The grey literature search was conducted in November 2019.

Title and abstract screenings were carried out by two reviewers with the web-based application Rayyan [28]. The full texts of potentially eligible publications were retrieved and screened against the inclusion criteria by two reviewers. Upon inclusion of full texts, the reference lists were hand-searched for further relevant literature. The authors of articles from the year 1990 onwards were contacted. Yet, no additional source of evidence and no further information on the type of HVAC appliances could be obtained. Relevant publications which were identified outside of the systematic search and also the primary sources of the systematic reviews were evaluated for eligibility. Duplicates were first identified with Endnote X9 (Clarivate Analytics, Boston/USA) and in a second step with Rayyan.

The data charting form (Additional file 3) was developed and tested with a sample of every different type of eligible publication identified. Charted data included the title, name of the authors, country of the first author, year and type of the study. The content was charted with the PICO scheme (Population, Intervention, Control, Outcome) where applicable. Additionally, publications were described qualitatively to capture all the relevant information regardless of study type. In this section, the field of application, addressed issues, problem statement and the findings of the publications were summarized. Data charting was performed by the first author and checked for completeness and accuracy by a second reviewer.

To delineate publications of HVAC use in medical treatment from publications only associated with HVAC use, the results are reported in two paragraphs. The first paragraph includes publications which report health effects of HVAC when used as a supportive element in medical treatment. Studies that reported the application of HVAC as part of a trial intervention were also included in this section. Publications were grouped in a short second paragraph if the HVAC system did not serve as intervention or no differentiation between air conditioning of the room and direct body cooling was made. All pieces of evidence are presented in two detailed tables. Conflicts regarding the inclusion of unusual HVAC designs or a fanning method were resolved by discussion within the reviewing team.

## Results

### Database search

**Fig. 1:**
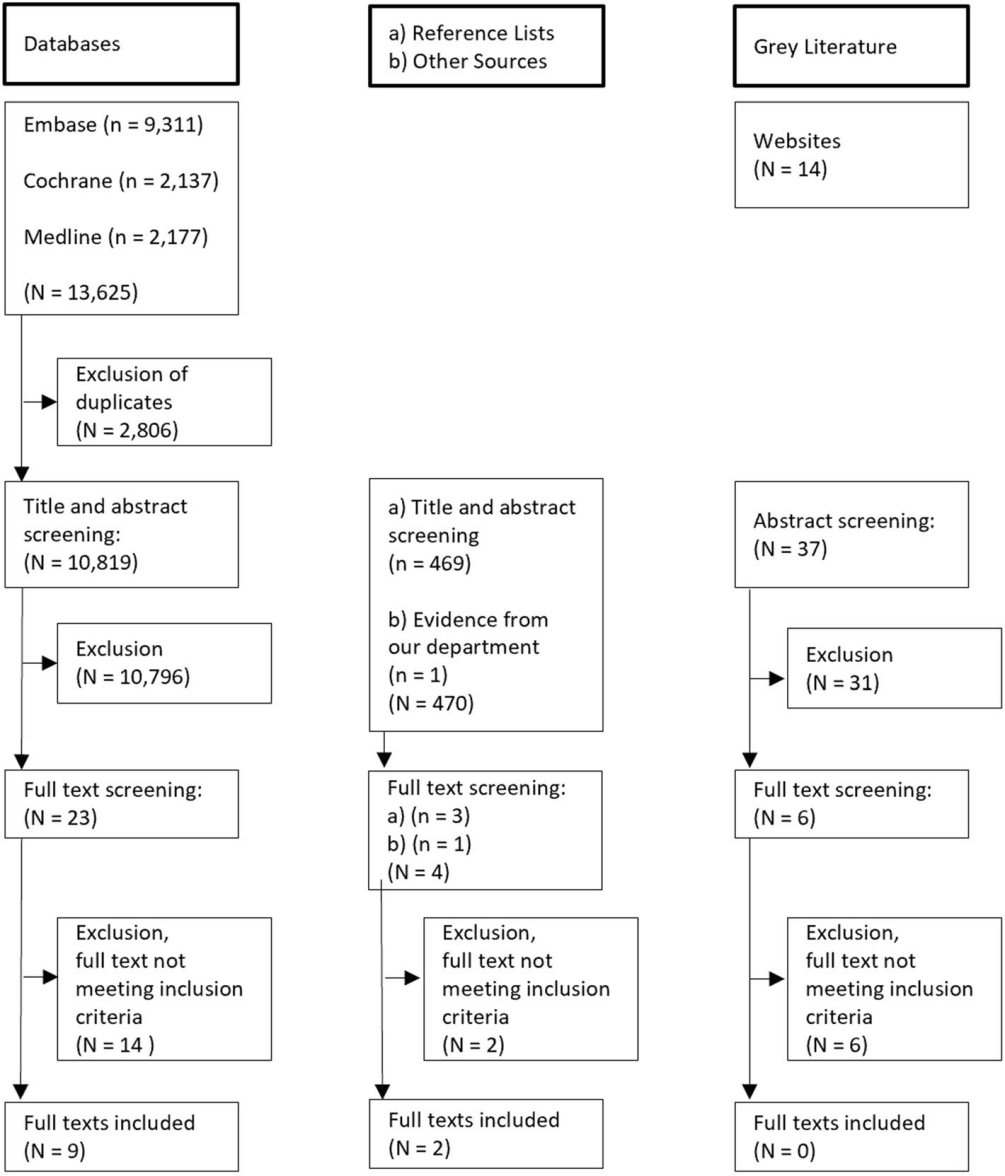
Flowchart of literature search

Database search resulted in 13,625 titles. After duplicates were removed, 10,819 titles and abstracts were screened, leading to the evaluation of 23 full texts. The only eligible systematic review retrieved by database search was not included because hand-searching of the primary sources revealed no eligible publications. Based on the database search, nine full texts were considered for analysis in this scoping review (cf. Figure 1).

### Reference lists, other sources and grey literature

Title and abstract screening of included publications and relevant other literature [25, 26, 29] yielded 469 publications of which one eligible publication was included. One additional abstract from our department was incorporated in the scoping review [30].

After searching an extensive set of websites for grey literature, only six full text publications matched the scope of this review and were checked for eligibility. None of these publications was selected for the analysis.

### HVAC use in the medical treatment

We identified seven publications reporting outcomes of HVAC use on patients which were published between 1959 and 2019 (cf. Table 1). This set consisted of five clinical trials [30–34], one cross-sectional study [35] and one case report [36]. Two publications were abstracts from conference proceedings [30, 34]. No study reported concrete measures for matching or randomisation of study participants. The studies originated from the USA (*n* = 3), UK (*n* = 1), Germany (*n* = 2) and France (*n* = 1).

**Table 1:** Studies on the health effects of various HVAC interventions for inpatients

Publication, Title	Type of Study	Objective	• **(I)** Intervention/ HVAC Specification (if given: mean temperature) • **(C)** Control (if given: mean temperature) • **(S)** Setting • **(D)** Duration of Intervention	Study Population	Health Effects of HVAC use/ Results/ Findings	Comments
Burch & DePasquale 1959, [31], “Influence of air conditioning on hospitalized patients”	Clinical trial & report of four exemplary cases	Analysis of the clinical course of patients depending on admission to an air-conditioned ward or non-air-conditioned ward	• **(I)** Ward no°1: Air conditioning (24 °C) • **(C)** Ward no°2: No air conditioning (30 °C) • **(S)** General ward • **(D)** Whole hospital stay	• Intervention: *n* = 88* • Control: *n* = 75*	Positive Effects: • Lower blood pressure & lower heart rate (statistically non-significant) • Favourable effects in 71 of 88 patients • Patients with congestive heart disease, asthma and chronic debilitation profited most • Easier nursing, reduction of odours, noise reduction (closed ward), improved morale • Cases 1–3: Improved walking, improved oedema, improved sleep, improved dyspnea, improved thermal comfort Negative Effects: • Reports of “getting a cold”, non-optimal thermal comfort, case 4: Development of a “cold”	• *Some patients were shifted between intervention groups • Only Afro-American women were included in the study • Various confounders are reported in the discussion
Burch & Hyman 1959, [32], “Influence of a hot and humid environment on the patient with coronary heart disease”	Clinical trial	Analysis of cardiac output/cardiac function either in a hot, non-air-conditioned ward or on an air-conditioned ward	• **(I)** Ward no°1: Air conditioning (23.3 °C) • **(C)** Ward no°2: No air conditioning (33.8 °C) • **(S)** General ward • **(D)** At least 1 h in each experimental condition	*N* = 5*	• Cardiac volume output and cardiac function were greater in all patients in the hot ward. Big individual differences were observed • Air-conditioned ward had a resting/recovery effect on the heart even if patients were acclimatised to hot weather conditions	• *All 5 patients were sequentially analysed in the hot and also in the air-conditioned ward -All patients were initially admitted to the hot ward − 2 patients were analysed first in the cold and subsequently in the hot ward − 3 patients were analysed in the hot ward and subsequently in the cold ward -Patients had at least 1 h to acclimatise on both wards • No patient with congestive heart failure was enrolled • No cardiological diagnosis was stated as a primary diagnosis for all patients
Carli et al. 1986, [33], “An investigation of factors affecting postoperative rewarming of adult patients”	Clinical trial	Analysis of the influence of a controlled environment in the recovery room on the rewarming rate of post-surgical patients	• **(I)** “Controlled ventilation and humidification system”, hospital 1, (22.9 °C) • **(C)** Regular recovery room without air conditioning, hospital 2, (22.4 °C) • **(S)** Post-surgery recovery room • **(D)** At least 1 h	• Intervention: *n* = 100 • Control: *n* = 100	• HVAC use had no significant influence on the rewarming rate • No difference in entrance or transfer to ward temperature was found • Age and type of anaesthesia were significant variables for the rewarming rate • Older patients rewarm slower after surgery than young patients • No difference found for shivering • No correlation between rewarming, duration of surgery and body fat	• No matching; allocation to intervention on a pragmatic basis • Only minor temperature difference between the two interventions • Exclusion of patients with endocrine abnormalities, extreme obesity and fever
Witt et al. 2018 [34], “Climate-controlled hospital patient rooms reduce indoor heat stress in patients with chronic obstructive pulmonary diseases and prevent an increased cardiorespiratory coupling”	Clinical trial	Analysis of the influence of an air-conditioned patient room on cardio-respiratory parameters in COPD patients	• **(I)** Regular patient room with convection free air conditioning (radiant cooling) (23 °C) • **(C)** Regular patient room without air conditioning (24.9° - 30.5 °C) • **(S)** General ward • **(D)** While 3-day heat waves	• Intervention: *n* = 13 • Control: *n* = 7	• Lower heart rate, lower respiratory rate • Increased heart rate variability • Decreased cardiorespiratory coupling	• No matching; allocation to intervention on a pragmatic basis • Only patients with COPD exacerbation were included
Witt et al. 2019 [30], “Accelerated patient recovery through improved indoor environment in hospital patient rooms - an adaptation strategy to urban heat in view of climate change”	Clinical trial	Analysis of the influence of an air-conditioned patient room on the clinical course, vital signs and mobility of patients with respiratory disease	• **(I)** Convection free air conditioning (radiant cooling) (23 °C) • **(C)** Regular patient room without air conditioning (15.9° - 28.2 °C) • **(S)** General ward • **(D)** Whole hospital stay	• Intervention: *n* = 63 • Control: *n* = 53	• Statistically significant reduction in length of stay (2 days) • Lower body temperature, lower diastolic blood pressure, lower fluid intake • Higher heart rate	• No matching; allocation to intervention on a pragmatic basis • Only patients with respiratory disease are included
Misset et al. 2006 [35], “Mortality of patients with heatstroke admitted to intensive care units during the 2003 heat wave in France: A national multiple-center risk-factor study”	Cross-sectional	Analysis of risk factors for positive or negative outcomes in heat stroke	• **(I)** Not fully applicable: Intensive care units with air conditioning* • **(C)** Not fully applicable: Intensive care units without air conditioning* • **(S)** Intensive care unit • **(D)** Whole intensive care unit stay	• Intervention: *n* = 158 • Control: *n* = 187	• Treatment of heat illness patients in wards without air conditioning leads to a 76% increased risk of death • Factors associated with survival of heat illness: Intensive care unit with air conditioning, psychiatric co-morbidity, use of antidepressant drugs, alcohol abuse	• The positive effect of air conditioning is not confounded by university status of hospitals, urban or rural location, number of beds and medical staff or number of admissions while study period • *Use of “external body cooling methods” was reported in all cases
Charoenpong et al. 2013, [36], “Complication of active rewarming in hypothermia from hypothyroidism”	Case report	Describing the clinical course and clinical adverse effects of rewarming a patient in the context of hypothermia due to hypothyroidism	• **(I)** Air conditioning (warm humidified air) and other methods • **(C)** Not applicable • **(S)** Intensive care unit • **(D)** Six hours	*N* = 1	• Rewarming was successful • Close monitoring for clinical deterioration is necessary	• The temperature of conditioned air was not specified

The reported HVAC systems ranged from conventional air conditioners to convection free air conditioning (radiant cooling) and fans. HVAC was implemented as a supportive element in the medical treatment with the goal of heat stress protection or as part of the rewarming strategy. HVAC was used on the general ward or the intensive care unit. The patient cohorts were comprised of patients with heat-stroke, hypothyroidism, pulmonary disease, diabetes mellitus, arthritis, goitre or patients who underwent intra-abdominal, pelvic, orthopaedic, peripheral vascular or prostatic surgery.

Regarding heat stress, the reported effects of cooling included improved blood pressure and respiratory rate and beneficial changes in cardiac function [30–32]. Particularly positive effects were observed in patients with chronic disease [31]. Notably, patients with congestive heart failure, coronary heart disease and asthma profited from the controlled room environment. Effects were described as a form of recovery effect from experiencing heat due to the use of air conditioning [30–32, 34]. The studies showed that equipping patient rooms with air conditioning led to earlier mobilisation [30, 31]. One study indicated a reduced length of hospital stay due to the use of radiant cooling [30]. Also, the protective effect of HVAC use in heat illness patients resulted in a reduction of mortality [35]. Only one study reported adverse effects [31]. Some patients perceived to get sick through air conditioning or described a negative impact on thermal comfort. However, the majority of patients and staff reported that air conditioning on the hospital ward was more beneficial to their overall well-being.

The use of HVAC in the recovery room showed no difference concerning the rewarming rate of postoperative patients [33]. Yet, the difference in room temperature between the two intervention groups was rather small (0.5 °C). HVAC utilisation was suggested as a supportive element in rewarming hypothyroid patients in one case report [36].

### Studies associated with HVAC use

We identified two clinical trials [37, 38] and two cross-sectional studies [39, 40] originating from the UK with relation to HVAC use, published between 1991 and 2018 (cf. Table 2). In these publications, either the application of the HVAC system could not be specified or no concrete health effects were reported. One publication was an abstract from a conference proceeding [40] and the remaining studies were journal articles.

**Table 2:** Studies associated with HVAC use

Publication, Title	Type of Study	Objective	• **(I)** Intervention/ HVAC Specification (if given: mean temperature) • **(C)** Control (if given: mean temperature) • **(S)** Setting • **(D)** Duration of Intervention	Study Population	Health Effects of HVAC Use/ Results/Findings	Comments
Price et al. 2003, [37], “Cooling strategies for patients with severe cerebral insult in ICU”	Clinical trial	Analysis of the efficacy of different cooling strategies for patients with cerebral insult and elevated body temperature	• **(I)** -Group a) paracetamol and depending on the clinical course: Continuation of paracetamol and **additional “fanning”** [portable rotary fans were preferably used] -Group b) no paracetamol and depending on the clinical course: Continuation with no intervention or **additional “fanning”** • **(C)** -Group a) paracetamol and depending on the clinical course: Continuation of paracetamol alone or paracetamol and additional other cooling methods (e.g. ice packs) • -Group b) no paracetamol and depending on the clinical course: Continuation with no intervention or use of other cooling methods (e.g. ice packs) • **(S)** Intensive care unit • **(D)** Criteria to end the procedure were not stated; it is referred to temperature measurements over 24 h	• *N* total = 67 Intervention: -Group a) paracetamol and fanning: *n* = 12 -Group b) no paracetamol and fanning: *n* = 16 Control: -Group a) paracetamol and another cooling strategy: *n* = 25 -Group b) no paracetamol and another cooling strategy: *n* = 14	• Physical cooling strategies (fanning) do not add to body cooling regardless of paracetamol use • Use of fan is negatively associated with a reduction in body temperature	• Report of hygiene problems associated with fan use • Uneven patient allocation to different cooling methods • “Fanning” could not be specified
Ferguson & Martin 1991, [38], “A study of skin temperatures, sweat rate and heat loss for burned patients”	Clinical trial	Analysis of the influence of the thermal environment on heat loss/ evaporation of patients with severe burns - Intensive care unit: Measurement of skin temperature of volunteers; measurement of wound temperatures, evaporation from burn wounds and sweating rates of burned patients - General ward: Measurement of sweating rates of patients	• **(I)** - Intensive care unit: Clean air unit: Small box with ultra-flow clean air system with uniform heated airflow (cf. 41) (28 °C – 38 °C); - General ward: Additional heating • **(C)** Not fully applicable • **(S)** Intensive care unit, general ward • **(D)** Few days/until wounds dried off	Intervention 1 (intensive care unit): *n* = 12, volunteers: *n* = 6 Intervention 2 (general ward), temperature not specified: *n* = 7	• During the first 3 days wound temperature was cooler than intact skin; after that time wound temperature was higher • Evaporation rate from wounds varied between 100 and 700g/m^2^/h • With a higher percentage of burned surface, room temperature could be higher without initiating sweating • Radiation and convection loss is lower in ICU (higher temperature). Losses from burn wounds were lower in the first few days (lower wound temperature). Overall convective losses: Similar in ICU and general ward.	• Difficult to identify participants in intervention groups • Healthy volunteers were enrolled • Clean air unit shows features of air conditioning but reported maximum room temperatures are outside the thermal comfort range
Johnston et al. 2006, [39], “Body temperature management after severe traumatic brain injury: methods and protocols used in the United Kingdom and Ireland”	Cross-sectional	Survey on “methods and protocols used in the management of body temperature in patients with severe TBI“	• **(I)** Convection fan and other methods • **(C)** Not fully applicable • **(S)** Intensive care unit • **(D)** Up to 48 h	• 33 centres	• Besides fans, a variety of methods is used for temperature management • Only 2 out of 33 intensive care units have a convection fan implemented as the first-line method • If the first-line method failed to lower body temperature, mostly a combination of direct body cooling methods was used • Fan use was mentioned as an adjunct to first-line treatment	• The survey was conducted with intensive care nurses • “Convection fan” could not be specified
De Vries & Feix 2018, [40], “Fever burden, septic screening, and cooling therapies in brain injury patients on a regional neurosciences intensive care unit”	Cross-sectional	Analysis of fever incidence and adherence to fever management protocols in the intensive care unit	• **(I)** “Cold air humidification”, “fan” and other physical and medical cooling methods • **(C)** Not applicable • **(S)** Intensive care unit • **(D)** Not specified	• *N* = 38	• Temperature burden in febrile patients was high • Besides screening for fever causes, 9 patients were treated with paracetamol alone, 9 patients had two or more cooling methods, 5 febrile patients were not treated • Outcomes according to different methods are not reported	• The analysis deals with adherence to fever treatment protocols • Most patients (*n* = 29) had a subarachnoid haemorrhage • Interventions are part of a bundle of cooling strategies including medical treatment (e.g. paracetamol) • Frequencies for use of “cold air humidification” or “fan” are not specified • “Fan” could not be specified

*TBI*: traumatic brain injury

The employment of HVAC was analysed in regard to the treatment of fever and fever combined with stroke or traumatic brain injury [37, 39, 40]. One study analysed the skin evaporation of patients with severe burns [39]. Fans, air conditioners, special air conditioning devices (clean air unit [41]) and other unspecified cooling methods were reported for the use in the intensive care unit.

## Discussion

This scoping review resulted in the collation of 11 eligible publications from 1959 to 2019, whereof seven reported the use of HVAC as part of the medical treatment. The reported objectives were heat stress protection, body temperature management and the rewarming of hypothermic patients. HVAC was used in intensive care units and on general wards. Findings included patients from surgery and internal medicine. The technical specifications involved air conditioners, radiant cooling systems, special appliances (clean air unit) and fans. When applied while experiencing heat, HVAC use resulted in the improvement of physiologic parameters and greater mobility of inpatients. Health effects were mediated by alleviation of heat stress and an increase of the physiologic adaptive capacity of acutely ill patients. Negative aspects that were described included unfavourable thermal comfort or the feeling of getting sick. Notably, a shorter duration of hospital treatment and reduction of mortality were reported.

There are several limitations as some studies had small patient samples [32, 34, 36, 38], shifted patients between intervention and control group [31], included only one ethnic race and sex [31] or reported supplementary cooling methods [35]. The risk of bias and confounding is high owing to the absence or short description of randomisation or matching. Above all, HVAC use cannot be blinded. We are aware that the search strategy of this scoping review has a focus on inpatients of internal medicine. Therefore findings pertaining to other medical disciplines might not be represented. It is plausible that many findings are important for other specialities as well. HVAC use could e.g. be a particularly strong contributor to successful treatment in such areas as psychiatry, where treatment is especially influenced by environmental factors. However, the inclusion of further search terms was limited by the data handling capacity. Also, omitting pediatric patients leaves out a vulnerable group. However, the unique physiology and the diverging medical conditions justify a separate review. Differentiation between direct body cooling and air conditioning of the room was not possible for three of the 11 publications. In one study, an air conditioning system with an experimental design was used (cf. Table 2).

### Implications for research

Despite abundant epidemiological findings confirming the adverse effects of heat on health, this scoping review shows that research on hospital adaptation and the individual treatment of inpatients is lacking. For future research projects, we propose to analyse the effects of HVAC

- on the physiology of inpatients experiencing heat or cold. The goal is to better understand the vulnerability of certain patient groups and to identify individuals that profit the most from a climate-controlled indoor environment.
- to clarify the relationship between thermal comfort and health effects. Therefore patient satisfaction should be included as an outcome. The studies should cover groups not considered to be at high-risk for climate-related adverse health effects, too.
- to determine optimal technical settings for different patient groups and various hospital areas within the existing hospital infrastructure.
- on the health system. Costs, energy consumption, the efficiency of the medical treatment and ergonomics for health care workers should be investigated.

Future trials should randomise participants to the intervention and control group and report the extent of exposure to HVAC. Furthermore, medical treatment has to be standardised and HVAC specifications, as well as the outdoor climatic conditions, should be reported.

## Conclusion

This scoping review provides valuable insight into the fields of application for HVAC and its health effects for inpatients. Due to the paucity of systematic research to date, no general recommendation for or against the use of HVAC for inpatients can be made. Publications on positive effects of heat stress protection for vulnerable groups are encouraging but more basic research regarding HVAC application is required for the development of evidence-based guidelines. The promising potential of HVAC to moderate climate change hazards has to be further explored.

## Supplementary information

**Additional file 1.** Full search strategy for databases.

**Additional file 2.** List of websites.

**Additional file 3.** Data charting form.

## Data Availability

The datasets used and/or analysed during the current study are available from the corresponding author on request.

## Abbreviations

HVAC: Heating, Ventilation and Air Conditioning
TBI: Traumatic Brain Injury
