# Supplementary material for: Health effects of heating, ventilation and air conditioning on hospital patients: a scoping review
Source: BMC Public Health. 2020 Aug 26;20:1287. doi: 10.1186/s12889-020-09358-1 (PMC7448359; doi:10.1186/s12889-020-09358-1)
Supplement: Supplementary file 1 — Additional file 1. Full search strategy for databases. [file 12889_2020_9358_MOESM1_ESM.doc]

Database: Embase Classic+Embase

--------------------------------------------------------------------------------

1 exp hospital patient/ (158487)

2 patient*.ab,kw,ti. (9321361)

3 inpatient*.ab,kw,ti. (159052)

4 (adolescent and institutioniali*ed).ab,kw,ti. (0)

5 (adolescent and hospitali*ed).ab,kw,ti. (1436)

6 1 or 2 or 3 or 4 or 5 (9364106)

7 exp air conditioning/ (30072)

8 (air and condition*).ab,kw,ti. (56572)

9 (radiant and cool*).ab,kw,ti. (225)

10 fan*.ab,kw,ti. (35221)

11 exp room ventilation/ (1471)

12 (room and ventilation).ab,kw,ti. (6769)

13 hvac.ab,kw,ti. (468)

14 (controlled and environment).ab,kw,ti. (23633)

15 7 or 8 or 9 or 10 or 11 or 12 or 13 or 14 (147038)

16 *vital sign/ (1031)

17 (vital and sign*).ab,kw,ti. (86469)

18 *heart rate/ (52473)

19 (heart and rate).ab,kw,ti. (309968)

20 *blood pressure/ (88037)

21 (blood and pressure).ab,kw,ti. (516604)

22 *breathing rate/ (3657)

23 (breathing and rate).ab,kw,ti. (20826)

24 (respiratory and rate).ab,kw,ti. (94207)

25 *body temperature/ (14174)

26 (body and temperature).ab,kw,ti. (62623)

27 16 or 17 or 18 or 19 or 20 or 21 or 22 or 23 or 24 or 25 or 26 (978840)

28 exp exercise test/ (83720)

29 (exercise and test*).ab,kw,ti. (113436)

30 exp spirometry/ (42168)

31 spirometry.ab,kw,ti. (29040)

32 *body plethysmography/ (802)

33 (body and plethysmography).ab,kw,ti. (5236)

34 (lung and volume and measurement*).ab,kw,ti. (12131)

35 *blood gas analysis/ (2962)

36 (blood and gas* and analysis).ab,kw,ti. (33537)

37 (lung and function and test*).ab,kw,ti. (40885)

38 (respiratory and function and test*).ab,kw,ti. (27725)

39 exp forced expiratory volume/ (66262)

40 (forced and expiratory and volume).ab,kw,ti. (25420)

41 *forced expiratory flow/ (437)

42 (forced and expiratory and flow).ab,kw,ti. (7917)

43 fev*1.ab,kw,ti. (57227)

44 *peak expiratory flow/ (1615)

45 (peak and expiratory and flow).ab,kw,ti. (10490)

46 pef.ab,kw,ti. (6778)

47 28 or 29 or 30 or 31 or 32 or 33 or 34 or 35 or 36 or 37 or 38 or 39 or 40 or 41 or 42 or 43 or 44 or 45 or 46 (347434)

48 *kidney function test/ (2782)

49 (kidney and function and test*).ab,kw,ti. (20927)

50 *creatinine blood level/ (2689)

51 (creatinine and blood and level).ab,kw,ti. (13974)

52 (serum and creatinine).ab,kw,ti. (89229)

53 exp creatinine clearance/ (30882)

54 (creatinine and clearance).ab,kw,ti. (31536)

55 exp glomerulus filtration rate/ (93936)

56 (glomerular and filtration and rate).ab,kw,ti. (59077)

57 gfr.ab,kw,ti. (34666)

58 48 or 49 or 50 or 51 or 52 or 53 or 54 or 55 or 56 or 57 (229436)

59 *sleep time/ (4896)

60 (sleep and time).ab,kw,ti. (56050)

61 *polysomnography/ (3374)

62 polysomnography.ab,kw,ti. (24423)

63 somnography.ab,kw,ti. (68)

64 actigraphy.ab,kw,ti. (6227)

65 *actimetry/ (1420)

66 actimetry.ab,kw,ti. (264)

67 59 or 60 or 61 or 62 or 63 or 64 or 65 or 66 (80101)

68 27 or 47 or 58 or 67 (1512751)

69 6 and 15 and 68 (9311)

************************************************************************

Database: Ovid MEDLINE

--------------------------------------------------------------------------------

1 exp adolescent, hospitalized/ (429)

2 (adolescent and hospitali*ed).ab,kw,ti. (879)

3 exp adolescent, institutionalized/ (126)

4 (adolescent and institutioniali*ed).ab,kw,ti. (0)

5 exp inpatients/ (19378)

6 inpatient*.ab,kw,ti. (96356)

7 patient*.ab,kw,ti. (6165544)

8 1 or 2 or 3 or 4 or 5 or 6 or 7 (6192940)

9 exp air conditioning/ (2574)

10 (air and condition*).ab,kw,ti. (37021)

11 exp ventilation/ (5452)

12 (room and ventilation).ab,kw,ti. (3822)

13 (radiant and cool*).ab,kw,ti. (160)

14 fan*.ab,kw,ti. (25718)

15 hvac.ab,kw,ti. (290)

16 (controlled and environment).ab,kw,ti. (18741)

17 9 or 10 or 11 or 12 or 13 or 14 or 15 or 16 (90033)

18 exp vital signs/ (396230)

19 (vital and sign*).ab,kw,ti. (54090)

20 exp blood pressure/ (280931)

21 (blood and pressure).ab,kw,ti. (336220)

22 exp body temperature/ (83773)

23 (body and temperature).ab,kw,ti. (44863)

24 exp heart rate/ (163588)

25 (heart and rate).ab,kw,ti. (201749)

26 exp respiratory rate/ (2408)

27 (respiratory and rate).ab,kw,ti. (58264)

28 (breathing and rate).ab,kw,ti. (12806)

29 18 or 19 or 20 or 21 or 22 or 23 or 24 or 25 or 26 or 27 or 28 (831607)

30 exp blood gas analysis/ (34855)

31 (blood and gas* and analysis).ab,kw,ti. (18773)

32 exp exercise test/ (61279)

33 (exercise and test*).ab,kw,ti. (73468)

34 exp lung volume measurements/ (35187)

35 (lung and volume and measurement*).ab,kw,ti. (7556)

36 exp plethysmography, whole body/ (1820)

37 (body and plethysmography).ab,kw,ti. (3216)

38 exp spirometry/ (21074)

39 spirometry.ab,kw,ti. (15956)

40 (lung and function and test*).ab,kw,ti. (19933)

41 (respiratory and function and test*).ab,kw,ti. (13596)

42 (peak and expiratory and flow).ab,kw,ti. (7894)

43 pef.ab,kw,ti. (4522)

44 (forced and expiratory and volume).ab,kw,ti. (18915)

45 (forced and expiratory and flow).ab,kw,ti. (5845)

46 fev*1.ab,kw,ti. (29727)

47 30 or 31 or 32 or 33 or 34 or 35 or 36 or 37 or 38 or 39 or 40 or 41 or 42 or 43 or 44 or 45 or 46 (257116)

48 *Kidney Function Tests/ (6241)

49 (kidney and function and test*).ab,kw,ti. (9947)

50 (serum and creatinine).ab,kw,ti. (55055)

51 (creatinine and blood and level).ab,kw,ti. (7714)

52 (creatinine and clearance).ab,kw,ti. (20982)

53 exp Glomerular Filtration Rate/ (41117)

54 (glomerular and filtration and rate).ab,kw,ti. (39782)

55 gfr.ab,kw,ti. (18137)

56 48 or 49 or 50 or 51 or 52 or 53 or 54 or 55 (131133)

57 (sleep and time).ab,kw,ti. (31206)

58 exp polysomnography/ (19305)

59 polysomnography.ab,kw,ti. (12780)

60 somnography.ab,kw,ti. (23)

61 exp actigraphy/ (3112)

62 actigraphy.ab,kw,ti. (3250)

63 actimetry.ab,kw,ti. (141)

64 57 or 58 or 59 or 60 or 61 or 62 or 63 (52324)

65 29 or 47 or 56 or 64 (1171637)

66 8 and 17 and 65 (2177)

************************************************************************

Database: Cochrane

--------------------------------------------------------------------------------

ID Search Hits

#1 MeSH descriptor: [Adolescent, Hospitalized] explode all trees 6

#2 (adolescent AND hospitali*ed):ti,ab,kw 1105

#3 MeSH descriptor: [Adolescent, Institutionalized] explode all trees 1

#4 (adolescent AND institutionali*ed):ti,ab,kw 70

#5 MeSH descriptor: [Inpatients] explode all trees 863

#6 (inpatient*):ti,ab,kw 15704

#7 (patient*):ti,ab,kw 862083

#8 #1 OR #2 OR #3 OR #4 OR #5 OR #6 OR #7 864610

#9 MeSH descriptor: [Air Conditioning] explode all trees 31

#10 (air AND condition*):ti,ab,kw 3498

#11 MeSH descriptor: [Ventilation] explode all trees 75

#12 (room AND ventilation):ti,ab,kw 1008

#13 (radiant AND cool*):ti,ab,kw 9

#14 (fan*):ti,ab,kw 2205

#15 (hvac):ti,ab,kw 6

#16 (controlled AND environment):ti,ab,kw 6802

#17 #9 OR #10 OR #11 OR #12 OR #13 OR #14 OR #15 OR #16 13243

#18 MeSH descriptor: [Vital Signs] explode all trees 35192

#19 (vital AND sign*):ti,ab,kw 17475

#20 MeSH descriptor: [Blood Pressure] this term only 25724

#21 (blood AND pressure):ti,ab,kw 93037

#22 MeSH descriptor: [Body Temperature] this term only 2123

#23 (body AND temperature):ti,ab,kw 7538

#24 MeSH descriptor: [Heart Rate] this term only 18344

#25 (heart AND rate):ti,ab,kw 66639

#26 MeSH descriptor: [Respiratory Rate] this term only 228

#27 (respiratory AND rate):ti,ab,kw 16916

#28 (breathing AND rate):ti,ab,kw 5961

#29 #18 OR #19 OR #20 OR #21 OR #22 OR #23 OR #24 OR #25 OR #26 OR #27 OR #28 154904

#30 MeSH descriptor: [Blood Gas Analysis] explode all trees 2096

#31 (blood AND gas* AND analysis):ti,ab,kw 10188

#32 MeSH descriptor: [Exercise Test] explode all trees 8133

#33 (exercise AND test):ti,ab,kw 27934

#34 MeSH descriptor: [Respiratory Function Tests] this term only 3657

#35 (respiratory AND function AND test*):ti,ab,kw 7730

#36 MeSH descriptor: [Lung Volume Measurements] this term only 448

#37 (lung AND volume AND measurement*):ti,ab,kw 2389

#38 MeSH descriptor: [Plethysmography, Whole Body] this term only 96

#39 (body AND plethysmography):ti,ab,kw 727

#40 MeSH descriptor: [Spirometry] this term only 1596

#41 (spirometry):ti,ab,kw 5762

#42 (lung AND function AND test*):ti,ab,kw 7941

#43 MeSH descriptor: [Forced Expiratory Flow Rates] this term only 202

#44 (forced AND expiratory AND flow):ti,ab,kw 3587

#45 MeSH descriptor: [Peak Expiratory Flow Rate] this term only 1533

#46 (peak AND expiratory AND flow):ti,ab,kw 4799

#47 (pef):ti,ab,kw 2112

#48 MeSH descriptor: [Forced Expiratory Volume] this term only 5075

#49 (forced AND expiratory AND volume):ti,ab,kw 12063

#50 (forced AND expiratory AND flow):ti,ab,kw 3587

#51 (fev*1):ti,ab,kw 11275

#52 #30 OR #31 OR #32 OR #33 OR #34 OR #35 OR #36 OR #37 OR #38 OR #39 OR #40 OR #41 50493

#53 #42 OR #43 OR #44 OR #45 OR #46 OR #47 OR #48 OR #49 OR #50 OR #51 23735

#54 #52 OR #53 64317

#55 MeSH descriptor: [Kidney Function Tests] this term only 1171

#56 (kidney AND function AND test*):ti,ab,kw 4584

#57 MeSH descriptor: [Glomerular Filtration Rate] this term only 2519

#58 (glomerular AND filtration AND rate*):ti,ab,kw 7813

#59 (gfr):ti,ab,kw 7019

#60 (serum AND creatinine):ti,ab,kw 11912

#61 (creatinine AND blood AND level):ti,ab,kw 8354

#62 (creatinine AND clearance):ti,ab,kw 5650

#63 #55 OR #56 OR #57 OR #58 OR #59 OR #60 OR #61 OR #62 28009

#64 (sleep and time):ti,ab,kw 10623

#65 MeSH descriptor: [Polysomnography] this term only 1727

#66 (polysomnography):ti,ab,kw 3622

#67 (somnography):ti,ab,kw 10

#68 MeSH descriptor: [Actigraphy] this term only 383

#69 (actigraphy):ti,ab,kw 1353

#70 (actimetry):ti,ab,kw 657

#71 #64 OR #65 OR #66 OR #67 OR #68 OR #69 OR #70 13443

#72 #29 OR #54 OR #63 OR #71 227171

#73 #8 AND #17 AND #72 2137

************************************************************************
