# Supplementary material for: Health effects of heating, ventilation and air conditioning on hospital patients: a scoping review
Source: BMC Public Health. 2020 Aug 26;20:1287. doi: 10.1186/s12889-020-09358-1 (PMC7448359; doi:10.1186/s12889-020-09358-1)
Supplement: Supplementary file 2 — Additional file 2. List of websites. [file 12889_2020_9358_MOESM2_ESM.docx]

1. World Health Organization, [www.who.int](http://www.who.int)
2. European Commission, ec.europa.eu
3. German Federal Ministry of Health, [www.bundesgesundheitsministerium.de](http://www.bundesgesundheitsministerium.de)
4. German Federal Ministry of Education and Research, [www.bmbf.de](http://www.bmbf.de)
5. Berlin Senate Chancellery, www.berlin.de
6. Center for Disease Control, [www.cdc.gov](http://www.cdc.gov)
7. Health Canada, [www.canada.ca/en/health-canada.html](http://www.canada.ca/en/health-canada.html)
8. Public Health England, www.gov.uk
9. The Intergovernmental Panel on Climate Change, www.ipcc.ch
10. World Meteorological Organization, [www.public.wmo.int](http://www.public.wmo.int)
11. International Network for Information on Ventilation and Energy Performance, [www.inive.org](http://www.inive.org)
12. American Society of Heating, Refrigerating and Air-Conditioning Engineers (ASHRAE), www.technologyportal.ashrae.org
13. Air Infiltration and Ventilation Centre, www.aivc.org
14. Center for health design, www.healthdesign.org
