# Supplementary material for: Health effects of heating, ventilation and air conditioning on hospital patients: a scoping review
Source: BMC Public Health. 2020 Aug 26;20:1287. doi: 10.1186/s12889-020-09358-1 (PMC7448359; doi:10.1186/s12889-020-09358-1)
Supplement: Supplementary file 3 — Additional file 3. Data charting form. [file 12889_2020_9358_MOESM3_ESM.docx]

Data charting: Source of evidence

| **Item-Number of Publication #** | **Title** | **Authors** | **Country of first Author** | **Source** | **Citation** | **Year** | **Type of Study** |
| --- | --- | --- | --- | --- | --- | --- | --- |
|  |  |  |  | 1. Journal 2. Poster 3. Conference Proceedings |  |  | 1. Meta Analysis 2. Systematic Reviews 3. Scoping Review 4. Randomised Controlled Trial 5. Cohort Study 6. Case Control Study 7. Cross sectional 8. Case Series, Case Report 9. Editorials 10. Reports /Survey 11. Other |

Data charting: PICO scheme

| **Population** | **Intervention** | **Duration of the Intervention** | **Control** | **Outcome** |
| --- | --- | --- | --- | --- |
| 1. Adult inpatient 2. Elderly patient/Geriatrics 3. Women giving birth/Women in childbed 4. Critical Care 5. Single Patient/ Case Report 6. Mixed: In and Outpatients | 1. Air Conditioning 2. Radiant Cooling 3. Electric Fan 4. HVAC 5. Controlled Environment/ Unspecific 6. Air Conditioning as Context Factor 7. Medication/Cold Infusions 8. Direct body cooling methods 9. Other | 1. Whole hospital stay 2. Intermitting 3. Before Discharge 4. Acute treatment 5. Long term treatment | 1. Air Conditioning 2. Radiant Cooling 3. Electric Fan 4. HVAC 5. Controlled Environment/ Unspecific 6. Air Conditioning as Context Factor 7. Medication/Cold Infusions 8. Direct body cooling methods 9. Other | 1. Respiratory Parameters 2. Vital Signs 3. Kidney Function 4. Sleep/Polygraphy 5. Body Temperature 6. Other |

Data charting: Qualitative Description

| **Field of application** | **Addressed Issues** | **Problem Statement** | **Methodological Philosophy** | **Research Method** | **Source of evidence matches clearly scope of this review "AC-intervention"** | **Findings (relations to past research)** | **Remarks** |
| --- | --- | --- | --- | --- | --- | --- | --- |
| 1. General Hospital Care 2. Intensive Care Unit 3. Specify other | What's analysed in this paper? | What's the context/ rationale? | 1. Quantitative? 2. Qualitative? | 1. Prosepective    1. Obser-vational    2. Inter-ventional 2. Retrospective . | 1. In line with Scope 2. Air Conditioning methods are involved in the context of the source of evidence or room cooling is not specified |  |  |
